# Spatiotemporal analysis of land surface temperature and land cover changes in Prešov city using downscaling approach and machine learning algorithms

**DOI:** 10.1007/s10661-024-13598-8

**Published:** 2025-01-03

**Authors:** Anton Uhrin, Katarína Onačillová

**Affiliations:** Institute of Geography, Faculty of Science, Pavol Jozef Šafárik University in Košice, Šrobárova 2, 04001 Košice, Slovak Republic

**Keywords:** Land surface temperature, Land cover, Downscaling, Urban heat island, Machine learning classifiers

## Abstract

In recent decades, global climate change and rapid urbanization have aggravated the urban heat island (UHI) effect, affecting the well-being of urban citizens. Although this significant phenomenon is more pronounced in larger metropolitan areas due to extensive impervious surfaces, small- and medium-sized cities also experience UHI effects, yet research on UHI in these cities is rare, emphasizing the importance of land surface temperature (LST) as a key parameter for studying UHI dynamics. Therefore, this paper focuses on the evaluation of LST and land cover (LC) changes in the city of Prešov, Slovakia, a typical medium-sized European city that has recently undergone significant LC changes. In this study, we use the relationship between Landsat-8/Landsat-9-derived LST and spectral indices Normalized Difference Built-Up Index (NDBI), Normalized Difference Vegetation Index (NDVI), Normalized Difference Water Index (NDWI) derived from Landsat-8/Landsat-9 and Sentinel-2 to downscale LST to 10 m. Two machine learning (ML) algorithms, support vector machine (SVM) and random forest (RF), are used to assess image classification and identify how different types and LC changes in selected years 2017, 2019, and 2023 affect the pattern of LST. The results show that several decisions made during the last decade, such as the construction of new urban fabrics and roads, caused the increase in LST. The LC change evaluation, based on the RF classification algorithm, achieved overall accuracies of 93.2% in 2017, 89.6% in 2019, and 91.5% in 2023, outperforming SVM by 0.8% in 2017 and 4.3% in 2023. This approach identifies UHI-prone areas with higher spatial resolution, helping urban planning mitigate the negative effects of increasing urban LSTs.

## Introduction

Urbanization and population growth result in an uneven distribution of surfaces that absorb substantial solar radiation (Purio et al., 2022), thus altering land cover (LC) dynamics. These changes affect critical processes such as heat transfer (Shamsaei et al., 2022), air circulation (Longley et al., 2004), precipitation patterns (Patil & Surawar, 2023; Steensen et al., 2022), and urban thermal environments (Fadhil et al., 2023). This leads to a transformation of the microclimate, resulting in significant temperature differences between urban and rural areas, known as the urban heat island (UHI) effect (Grigoraș & Urițescu, 2019). Studies have shown that even in small- and medium-sized cities, factors such as urbanization, reduced vegetation, and increased human activity contribute to elevated temperatures compared to surrounding rural areas. Despite the significant impact of UHI on local climates, energy consumption, and public health in these smaller cities, research in this area remains limited, though several studies have attempted to address this gap (Miles et al., 2023; Ivajnšič & Žiberna, 2018; Vardoulakis et al., 2013; Dobrovolný, 2013).

A systematic review by Almeida et al. (2021) identifies a significant increase in UHI research in recent years, particularly those utilizing remote sensing techniques. These studies are crucial, as they provide insights into how negative anthropogenic processes—such as population growth, urbanization, and land cover changes—affect local climates. This reinforces the importance of UHI research not only for the scientific community but also for policymakers and the general public. Remote sensing, in particular, facilitates the development of region-specific solutions by providing essential data on localized temperature patterns and urbanization impacts.

Despite this growing body of research, detailed studies on UHI effects in medium-sized cities remain limited. These cities exhibit unique urban dynamics, such as distinct land-use patterns and growth characteristics, requiring tailored approaches for effective UHI mitigation strategies. Previous studies have predominantly focused on UHI effects in large metropolitan areas, leaving a gap in research on medium-sized cities. However, these cities are crucial for understanding localized UHI dynamics due to their specific land-use and urbanization characteristics.

For instance, medium-sized cities in Europe, such as Bolzano, Bratislava, Nice, Rijeka, Porto, Stavanger, and Trnava, demonstrate unique thermal profiles influenced by land-use configurations and urban planning practices (Hellings & Rienow, 2021; Holec et al., 2020). Urban land-use patterns—such as the extent of impervious surfaces, green spaces, and building density—play a significant role in shaping local climate conditions.

Urban planning practices, including the distribution of green spaces and the management of built-up areas, also affect the severity of the UHI effect. For instance, a study of diurnal variability in air temperature patterns across five Central European cities (Bratislava, Brno, Kraków, Szeged, and Vienna) during the 2015 heatwave revealed significant spatial variability influenced by urban land use, land cover (LU/LC), and relief features (Bokwa et al., 2019). These localized findings highlight the importance of UHI research in medium-sized cities to develop tailored climate adaptation strategies. As these cities house a substantial portion of the global urban population, their vulnerability to UHI and its impacts on public health and energy consumption necessitate urgent attention.

Medium-sized cities, characterized by populations ranging from 100,000 to 500,000, collectively host nearly half of the world’s urban population (United Nations, 2015). This significant concentration of urban dwellers highlights the critical need for localized UHI research to address the unique challenges and dynamics of these cities. Prešov serves as a particularly relevant case study for examining UHI effects in medium-sized cities. Its distinct urbanization patterns and growth dynamics provide valuable insights into the challenges and opportunities for managing UHI impacts in similar urban contexts.

Studying the UHI effect relies heavily on analyzing LST data, which provides critical insights into spatial and temporal variations in surface temperatures. This understanding is essential to assess urban heat fluxes, identify the presence and intensity of the UHI phenomenon (Han et al., 2023; Tanoori et al., 2024), simulate surface energy exchange (Khan et al., 2023), and study the energy balance of the surface at global, regional, and local scales (Yan et al., 2024). LST is also widely used to assess surface moisture and drought levels (Weiland et al., 2023; Xiong et al., 2022).

A robust and efficient method for acquiring LST data involves the use of freely available thermal satellite imagery, facilitating a comprehensive analysis of temperature patterns across broad geographic regions. In urban environments, the spatial and temporal heterogeneity of surface temperature is pronounced due to complex surface structures and materials, reflecting the diversity of urban LC. However, freely available data from thermal sensors typically have coarser spatial resolution and higher temporal resolution, which is inadequate for accurately identifying thermal characteristics of urban areas and conducting detailed analyses of factors influencing the UHI formation (Song & Park, 2020). The resolution of acquired thermal data varies, ranging from 1000 m for AVHRR to 100 m for Landsat-8/Landsat-9 (Cheval et al., 2011) and 90 m for the Aster sensor onboard the Terra satellite (Yamaguchi et al., 1998), making it more suitable for global to regional scale studies.

Advancements in satellite technology, improvements in multispectral and thermal sensors, rapid development of GIS, and remote sensing methods, along with the availability of open-source platforms such as Google Earth Engine (GEE), are crucial for overcoming these limitations and enabling higher-resolution LST mapping. One of the most widely used approaches to achieve higher spatial resolution in satellite imagery for deriving LST is downscaling methods, transforming a coarser spatial resolution image into a higher spatial resolution image (Hu et al., 2024). There are several downscaling approaches or models that play a role in facilitating LST analyses and reducing computational time and storage space (Hutengs & Vohland, 2016; Xu et al., 2021).

This work offers one of the first investigations into a complex spatiotemporal analysis of recent LC and LST changes in the city of Prešov, which has been not the subject of UHI studies before (to date) on the local scale for the years 2017, 2019, and 2023 with the use of an open-source cloud platform GEE. We derive LST within the urban environment of the Prešov city at higher spatial resolution using downscaling of Landsat thermal satellite data with multispectral indices derived from Sentinel 2 data. We also use semi-automatic classification of satellite data (Sentinel-2) using selected machine learning algorithms—support vector machine and random forest. Information about LST of different urban surfaces and land use types can help to suggest effective strategies in the areas of efficient urbanization, urban planning, or sustainable development proposals to minimize the negative impacts of climate change and UHIs to optimize or enhance the quality of life of urban residents.

## Study area

**Fig. 1:**
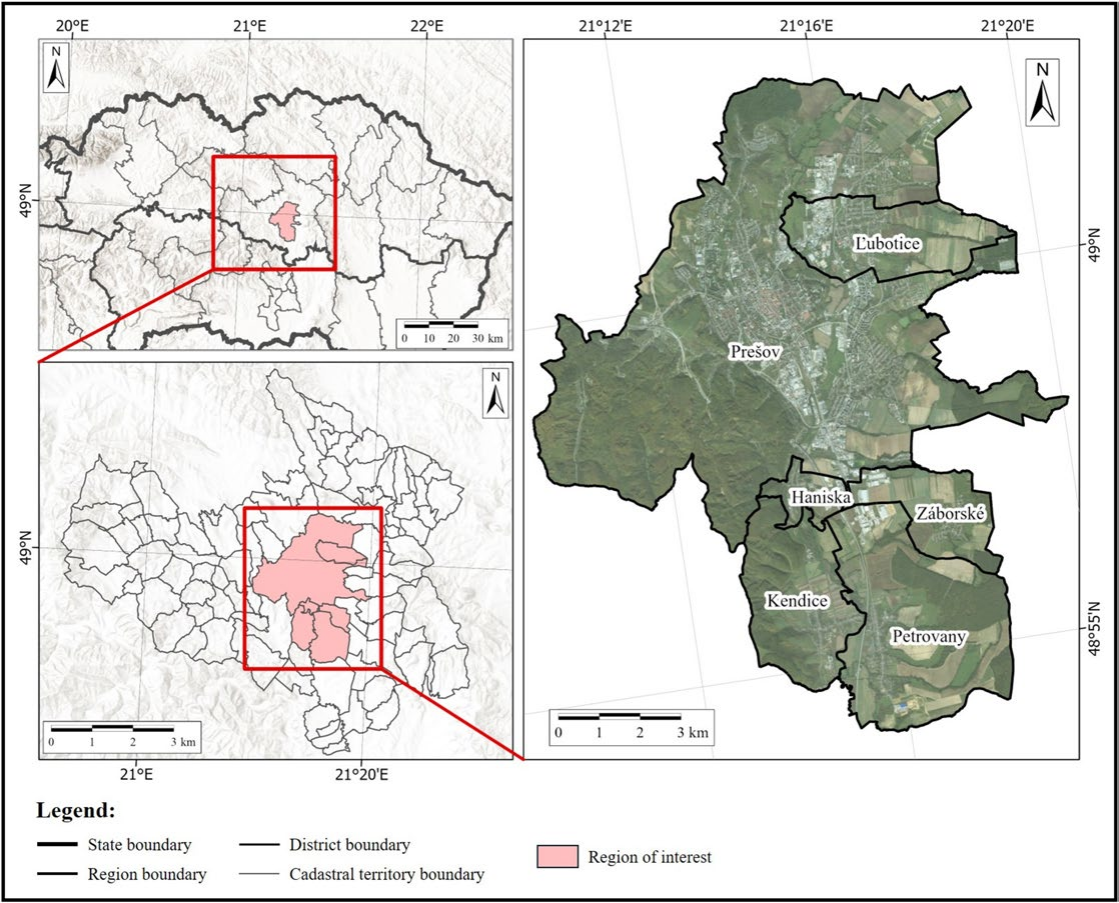
Location of the study area of the Prešov city. The background maps are © ESRI. “World Imagery” and “Terrain with labels,” acquired on March 7, 2024

The area of interest is the medium-sized city of Prešov with the adjacent municipalities of Haniska, Kendice, Ľubotice, Petrovany, and Záborské, located in eastern Slovakia and characterized by a typical Central European urban environment with significant heterogeneity of LC classes (Fig. 1). The total area of the city is approximately 70.44 km^2^ with a total population of 84,824 inhabitants, which represents approximately 1204.20 inhabitants per 1 km^2^ (Statistical Office SR, 2022).

Agricultural land occupies 35%, and forests account for 31%. Artificial surfaces are also highly represented, mainly by built-up areas that occupy 28%—continuous urban fabric accounts for 4%, discontinuous urban fabric accounts for 8%, and more than 7% are occupied by industrial, commercial, public, military, and private transport units. The road and rail network and associated land account for less than 4%. Sports and leisure facilities comprise 2% and green urban areas account for 1.5% (Copernicus Land Monitoring Service, 2018).

According to Köppen’s climate classification (Köppen & Geiger, 1936), the territory of Prešov lies in the mild continental climate zone, classification subtype “Dfb,” which is characterized by significant seasonality, with warm and humid summers, severe winters, and short dry periods (Kottek et al., 2006). The largest watercourse that flows through the territory of the city is the Torysa River and its left-hand tributaries Sekčov and Delňa.

## Materials and methods

The research in this study uses multispectral imagery collected by two satellite missions—the Landsat-8/Landsat-9 mission by NASA and the Sentinel-2 mission by ESA. Six multispectral satellite products obtained over the Prešov area were employed for selected days in 2017, 2019, and 2023: Landsat-8 OLI/TIRS sensor (3 products) and MSI sensor onboard the Sentinel-2 satellite (3 products). These six satellite Landsat-8/9 and Sentinel-2 data products were selected based on the proximity of their acquisition dates within the same year, considering similar atmospheric conditions and the lowest possible percentage of cloud cover. For our analysis, we selected only images with near 0% cloud cover over the city (although the scene cloud cover values in Table 1 may be higher, these values represent the entire scene, which encompasses a much larger area, while the Prešov area of interest remained cloud-free despite higher overall cloud cover reported). The corresponding air temperature parameters extracted from the OGIMET service developed by the Spanish Meteorological Institute (www.ogimet.com) are stated in Table 1.

**Table 1:** Selected parameters of the satellite products and the corresponding air temperature based on meteorological data from SHMÚ (2024)

Satellite, sensor and product	Product name	Acquisition date	Acquisition time (UTC)	Scene cloud cover (%)	Air temperature (°C)		
					09:00	10:00	
Landsat-8 OLI/TIRS L2 C2	LC08_L1TP_187026_20170811_20200903_02_T1	11.08.2017	09:26:41	0.42	29.7		30.8
Landsat-8 OLI/TIRS L2 C2	LC08_L1TP_187026_20190630_20200827_02_T1	30.06.2019	09:26:34	0.02	25.4		27.3
Landsat-9 OLI/TIRS L2 C2	LC09_L1TP_187026_20230719_20230719_02_T1	19.07.2023	09:26:16	12.61	25.8		26.9
Sentinel-2A MSI L2A	S2A_MSIL2A_20170803T094031_N0205_R036_T34UEV_20170803T094046	03.08.2017	09:40:46	6.04	29.6		30.7
Sentinel-2A MSI L2A	S2A_MSIL2A_20190701T093041_N0212_R136_T34UEV_20190701T115615	01.07.2019	09:30:39	6.59	27.6		28.9
Sentinel-2B MSI L2A	S2B_MSIL2A_20230718T093549_N0509_R036_T34UEV_20230718T112309	18.07.2023	09:35:49	11.55	20.9		22.3

Three USGS Landsat-8/Landsat-9 Level-2 Collection-2 Surface Reflectance products were used to derive three selected spectral indices that are closely related to LST at a spatial resolution of 30 m and to calculate atmospherically corrected LST itself using a modified radiative transfer equation inversion methodology (Table 1):

“LC08_L1TP_187026_20170811_20200903_02_T1” (11/08/2017, 09:26:41 UTC) for 2017

“LC08_L1TP_187026_20190630_20200827_02_T1” (30/06/2019, 09:26:34 UTC) for 2019

“LC09_L1TP_187026_20230719_20230719_02_T1” (2023/07/19, 09:26:16 UTC) for 2023

Three Harmonized Sentinel-2 MSI: MultiSpectral Instrument, Level-2A products were used to obtain the same spectral indices as in the case of the Landsat-8 satellite, but in a higher, 10-m spatial resolution, which has been used in the downscaling process. The products were selected based on the proximity of the acquisition date to the selected products of the Landsat-8/Landsat-9 satellite:

“S2A_MSIL2A_20170803T094031_N0205_R036_T34UEV_20170803T094046” (08/03/2017, 09:40:46 UTC) for 2017

“S2A_MSIL2A_20190701T093041_N0212_R136_T34UEV_20190701T115615” (07/01/2019, 09:30:39 UTC) for 2019

“S2B_MSIL2A_20230718T093549_N0509_R036_T34UEV_20230718T112309” (2023-07-18, 09:35:49 UTC) for 2023

Level-2A products provide users with orthorectified bottom-of-atmosphere surface reflectance, the classification of the scene, including cloud cover and cloud shadows, aerosol optical thickness, and water vapor maps (Gascon et al., 2017).

**Fig. 2:**
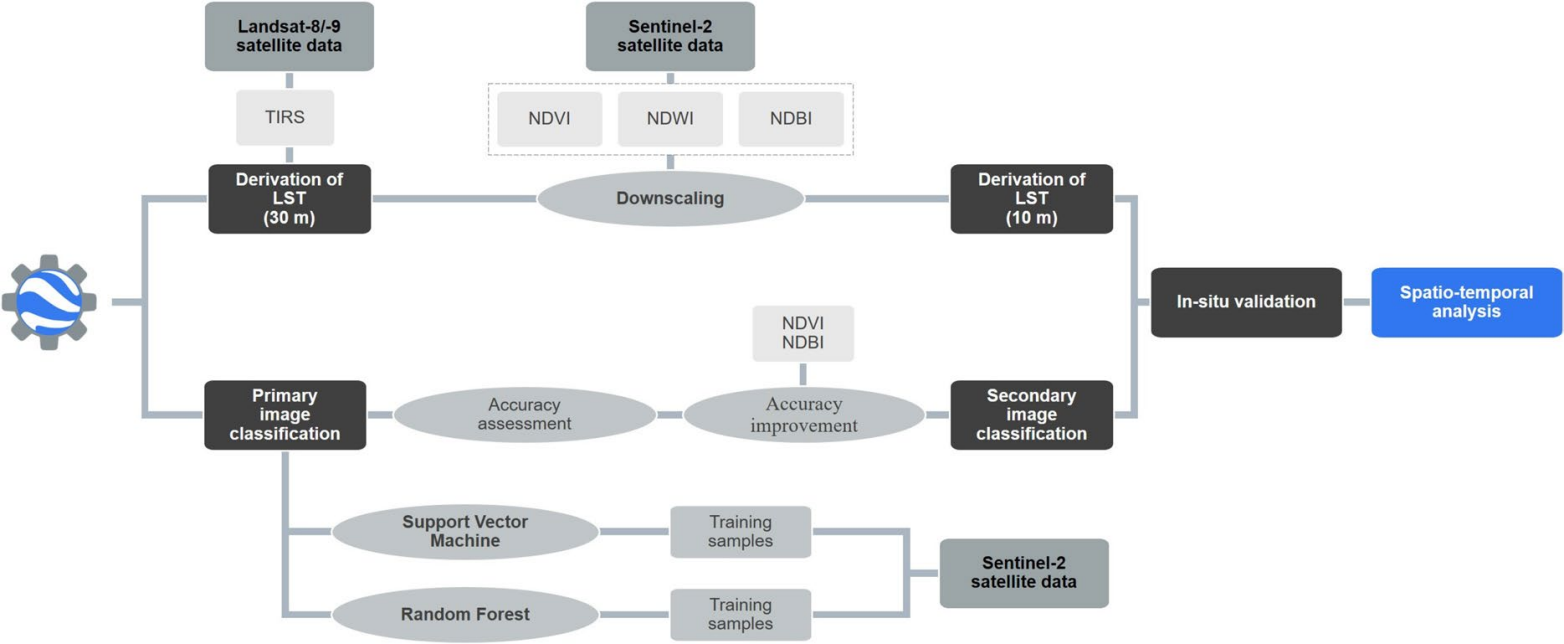
Flowchart summarizing the basic steps of land surface temperature downscaling and image classification using machine learning algorithms

The workflow is fully coded in JavaScript using the Code Editor Platform of the online cloud application GEE. The multispectral imagery was processed using the pipeline shown in Fig. 2, and the script itself is divided into two main parts: processes associated with the LST downscaling approach and processes of semi-automatic image classification using machine learning algorithms, which are described in detail in the subsections below.

### Validation data

**Fig. 3:**
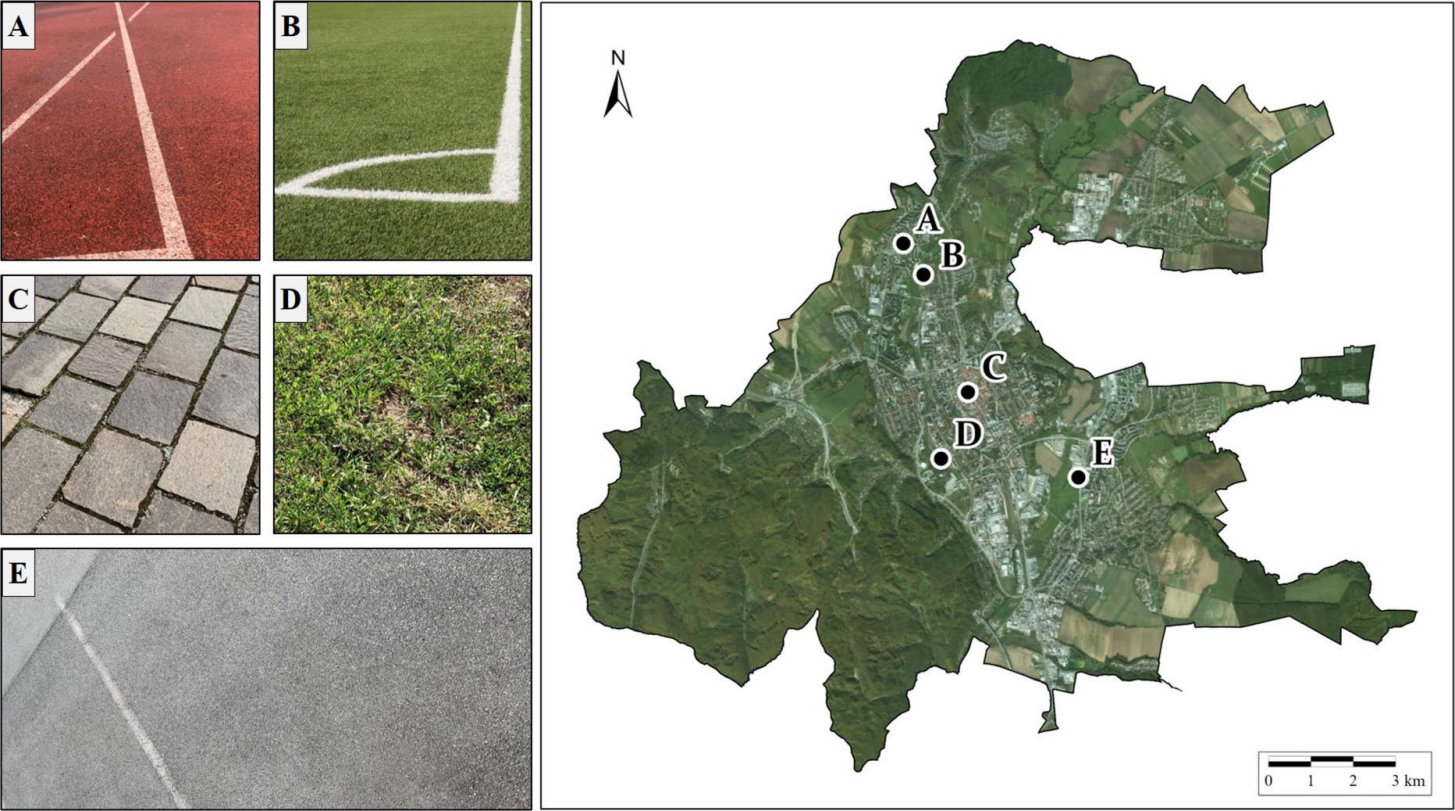
Five localities, which were the subject of field validation measurements within the area of interest: **A** tartan, (**B**) artificial grass, (**C**) stone paving, (**D**) grass, and (**E**) concrete; source: photos (**A**–**E**) author ‘s archive, background maps are © ESRI. “World Imagery,” acquired on August 23, 2023

To validate the LST downscaling results, field measurements of surface kinetic temperature obtained by temperature probe Pt1000TG7/E with a Comet data logger (as in Hofierka et al., 2020) were used at five selected open-sky localities within the study area to ensure their reliable identification even in satellite images and to represent various types of surfaces, as shown in Fig. 3.

Two natural materials—stone pavement (Fig. 3C) and grass (Fig. 3D)—and three artificial surfaces—tartan (Fig. 3A), artificial grass (Fig. 3B), and concrete (Fig. 3E)—were selected. The device allows measurement of the surface temperature in 5-s measurement intervals in the range of − 30 to 200 °C with a measurement accuracy of 0.15 °C (TR Instruments, 2023). Validation measurements were carried out in the area of interest on 19 July 2023 at 7:00 a.m. and 10:00 a.m. to match the acquisition time of the Landsat-9 satellite on the same day, whose thermal data were used to derive LST (acquisition time 09:26:16 UTC).

## LST downscaling methodology

Landsat-8/Landsat-9 TIRS allows for thermal radiation to be mapped at a native 100-m spatial resolution, which is resampled by the United States Geological Survey (USGS) to 30 m for consistency with other spectral bands of these satellites. Thermal radiation can be later converted to LST; however, for detailed analysis of LSTs in heterogeneous urban areas, the coarse spatial resolution of 30 m and longer intervals between acquisitions of these satellites are not sufficient to capture interactions of LST in terms of the heterogeneous character of LC. On the contrary, Sentinel-2 does not have a thermal band, so it is not possible to directly derive LST. However, due to its high spatial resolution (native 10-m resolution in visible and near-infrared (NIR) bands) and temporal resolution (recording every 6 days), this satellite has a high potential for calculating various spectral indices, allowing us to accurately capture the complexity of urban LC in better detail compared to the Landsat-8/Landsat-9 satellites. In this study, the spatial downscaling approach involves establishing a multiple linear model utilizing selected three spectral indices and LST obtained from the thermal band of the Landsat-8/Landsat-9 satellite according to the model adopted from Bonafoni et al. (2016) and Onačillová et al. (2022). The proposed model is capable to predict LSTs in 10-m spatial resolution by using spectral indices obtained from Sentinel-2 satellite data as input.

### Calculation of LST predictors—spectral indices

The first step involved preparation of LST predictors: three spectral indices—Normalized Difference Vegetation Index, NDVI (Guo et al., 2021; Purevdorj et al., 1998); Normalized Difference Built-Up Index, NDBI (Goldblatt et al., 2018; Zha et al., 2003); and Normalized Difference Water Index, NDWI (Camps et al., 2020; McFeeters, 1996)—were chosen and derived from Landsat-8/Landsat-9 satellite images with a 30-m spatial resolution and Sentinel-2 satellite images with a 10-m spatial resolution. The formulas for calculating the selected spectral indices of the Landsat-8/9 and Sentinel-2 satellites are stated in Table 2.

**Table 2:** Spectral indices used for land surface temperature downscaling

Acronym	Description	Formulation	Modified formulation for Landsat-8/Landsat-9 bands	Modified formulation for Sentinel-2 bands
NDVI	Normalized difference vegetation index	(NIR − RED/(NIR + RED)	(B5 − B4)/(B5 + B4)	(B8 − B4)/(B8 + B4)
NDWI	Normalized difference water index	(Green − NIR)/(Green + NIR)	(B3 − B5)/(B3 + B5)	(B3 − B8)/(B3 + B8)
NDBI	Normalized difference built-up index	(SWIR1 − NIR)/(SWIR1 + NIR)	(B6 − B5)/(B6 + B5)	(B11 − B8)/(B11 + B8)

The use of spectral indices such as NDVI, NDWI, and NDBI is well-established in remote sensing for their ability to characterize key land surface features that influence urban microclimae and LST variations. NDVI, a widely used index for monitoring vegetation cover, typically shows a negative correlation with LST, as denser vegetation is associated with cooler surface temperatures (Purevdorj et al., 1998). NDWI is primarily employed for identifying and delineating water bodies, which have a cooling effect on the surrounding environment (Gao, 1996). NDBI is commonly applied to identify built-up areas, which generally exhibit higher temperatures due to the absorption and retention of heat (Zha et al., 2003). Collectively, these indices enable the identification of different land surface types, including vegetated, water-covered, and built-up areas, which are key factors in driving UHI effects and influencing LST dynamics in heterogeneous urban environments. These indices are therefore integral to downscaling LST and understanding the spatial variability of LST patterns in urban landscapes.

Several studies, including Guha et al. (2022), Govil et al. (2019), and Chen and Zhang (2017), have demonstrated the effectiveness of using indices like NDVI, NDBI, and NDWI to estimate LST across heterogeneous urban areas. These studies highlight the complementary strengths of these indices in capturing diverse land surface features and their thermal dynamics, providing a comprehensive framework for analyzing and managing urban thermal environments.

**Fig. 4:**
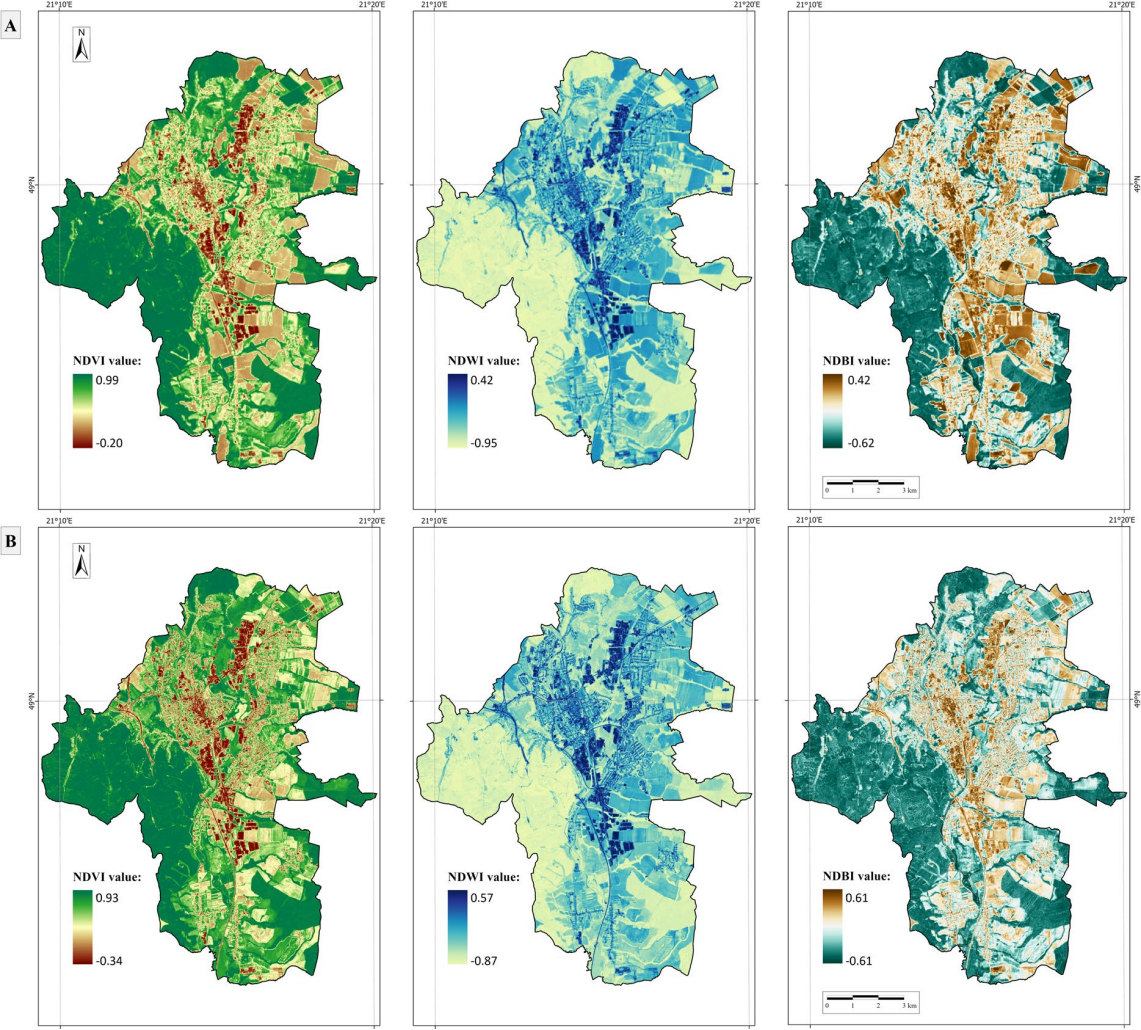
Normalized Difference Vegetation Index (NDVI), Normalized Difference Water Index (NDWI), and Normalized Difference Build-Up Index (NDBI) spectral indices derived from (**A**) Landsat-9 OLI satellite data in 30-m spatial resolution for 19 July 2023, (**B**) Sentinel-2 MSI satellite data in 10-m spatial resolution for 18 July 2023, for the area of interest encompassing the city of Prešov and surrounding municipalities

To derive NDVI from the Landsat-8/9 satellite, the NIR band with wavelengths of 0.85–0.88 μm and the red (R) band at wavelengths of 0.64–0.67 μm were used. The input spectral bands for the NDWI derivation were the green band with wavelengths of 0.533–0.590 μm and the NIR band. For the NDBI derivation, these were the NIR and shortwave infrared 1 (SWIR1) bands with a wavelength of 1.57–1.65 μm. All these bands had a native 30-m spatial resolution (Fig. 4A).

Equivalent spectral indices were derived from Sentinel-2 satellite data: the NIR band with a central wavelength of 0.84 μm and the red band with a central wavelength of 0.66 μm were used to calculate the NDVI. The green band with a central wavelength of 0.56 μm and the NIR band were used to obtain the NDWI. NDBI was calculated based on the NIR and SWIR1 bands with a central wavelength of 1.61 μm (resampled from the native spatial resolution of 20 to 10 m to ensure consistency with the resolution of NDVI and NDWI) (Fig. 4B).

### Multiple linear regression model between LC indices and LST

In this study, the spatial downscaling approach relies on establishing a linear relation utilizing three spectral indices derived from Landsat-8/9 imagery (NDVI, NDWI, NDBI) as predictors, with LST serving as the predicted variable. Then the established model is applied to the study areas with finer resolution. The LST with a spatial resolution of 30 m was derived from the surface temperature (ST_B10) of the Landsat-8/9 TIRS sensor. ST_B10 is derived from Landsat-8 Collection 2 Level 1 Thermal Infrared Sensor (TIRS) Band 10 data (USGS, 2024).

Then, the multiple linear regression statistical model between LST and spectral indices was constructed as predictors at a coarser (30 m) resolution. The model for the calculation of LST at the coarser level from Landsat-8/Landsat-9 imagery, using the output regression coefficients to calculate the LST in 30-m spatial resolution, is expressed in Eq. (1).

1 $${LST}_{c}={a}_{0}+ {a}_{1}\times {NDVI}_{c}+ {a}_{2}\times {NDBI}_{c}+ {a}_{3}\times {NDWI}_{c}$$

where the subscript *c* means the variable with coarser 30-m spatial resolution derived from Landsat-8/Landsat-9 satellite data and the variables a_0_–a_3_ represent the output regression coefficients.

Then, these derived regression coefficients are applied to obtain the downscaled LST using selected Sentinel-2 NDVI, NDBI, and NDWI spectral indices with finer 10-m spatial resolution, as shown in Eq. (2). The fundamental premise of this method is that the thermal bands have a direct relation with the spectral bands, and the relationship is scale invariable (Gao et al., 2012). The subsequent model was employed to downscale LST at finer resolution.

2 $${LST}_{f}={a}_{0}+ {a}_{1}\times {NDVI}_{f}+ {a}_{2}\times {NDBI}_{f}+ {a}_{3}\times {NDWI}_{f} + \Delta {LST}_{f}$$

where the coefficients a_0_–a_3_ of Eq. (1) are applied and the subscript *f* means the variable (spectral index) with a finer 10-m spatial resolution derived from Sentinel-2 satellite imagery.

The final LST_f_ contains regression residuals ∆LST_f_ added back to the downscaled map. In this way, the original, coarse-temperature field is recovered through reaggregation, and the spatial variability of the LST that depends on factors other than the predictors employed is taken into account. The grid of regression residuals is first calculated by subtracting the observed LST_obs_ from the modeled LST_c_ at 30 resolution (from Eq. (1) (Eq. 3). ∆LST_c_ is then resampled to ∆LST_f_ as a 10-m cell-size grid by convolution with a Gaussian kernel of 30-m size:

3 $$\Delta {LST}_{c}={LST}_{c}-{LST}_{obs}$$

The downscaling process employs a linear regression model, chosen for its robustness and ability to efficiently capture the relationships between LST and predictor variables (e.g., NDVI, NDBI, and NDWI). Linear regression is computationally efficient, interpretable, and particularly well suited for scenarios where predictor variables exhibit linear or near-linear correlations with the dependent variable, as demonstrated in several studies (e.g., Bonafoni et al., 2016; Chen & Zhang, 2017; Guha et al., 2022). Its application in this context allows for effective spatial scaling by linking coarse-resolution LST data to finer-scale land surface characteristics. This approach enables a detailed and reliable representation of spatial thermal patterns by integrating land surface characteristics, making it a valuable tool for addressing the complexity of urban environments.

## Image classification using machine learning algorithms

Support vector machine (SVM) and random forest (RF) algorithms were used and compared for the image classification of multispectral imagery using the “Harmonized Sentinel-2 MSI level 2A” data product.

Firstly, three cloud-free median compositions were generated for the respective years 2017, 2019, and 2023. This process involved utilizing Sentinel-2 satellite scenes that met specific criteria: captured between June 1st and August 8th of each selected year with cloud cover below 20%. Subsequently, these composites were displayed as true color RGB compositions used to create five datasets of training samples, representing the five dominant classes/types of classified LC—water bodies, forests, grasslands, agricultural lands, and built-up areas—which were systematically distributed across our study area. These training samples were generated separately for the years 2017, 2019, and 2023 to accurately capture the respective types of LC for each year. Subsequently, these training samples were used for image classification using the SVM and RF algorithms.

In GEE, the SVM algorithm implemented under the “ee.Classifier.libsvm()” tool was applied. An overview of the parameters and their respective values used to train the SVM model is presented in Table 3. The SVM classifier was configured with parameters optimized for the specific requirements of the study. The kernel type was set to linear, chosen for its computational efficiency and suitability for linearly separable data. This configuration facilitates straightforward and interpretable decision boundaries. The SVM type was set to C-SVC, optimized for multi-class classification tasks. Additionally, the cost parameter (C), which controls the trade-off between achieving a low error on the training data and maximizing the margin between the classes, is set to 10. This value is selected based on preliminary experiments to balance model complexity and classification accuracy. The choice of a linear kernel and the cost parameter helps achieve a model that is both computationally efficient and suitable for the relatively high-dimensional spectral data in this study.

**Table 3:** Parameters used for image classification by support vector machine and random forest algorithms in the Google Earth Engine environment. Source: Google Earth Engine (2024) author’s processing

Algorithm	Parameter	Value
SVM	Decision procedure	Default, voting
	SVM type	Default, C_SVC (for non-linear data)
	Kernel type	Linear
	Cost	10
RF	Number of decision trees	1500
	Number of variables per split	Default, the square root of the number of variables
	Minimum leaf population	Default, 1
	Randomization seed	Default, 0
	Fraction of input to bag per tree	Default, 0.5
	Maximum number of leaf nodes	Default, no limit

RF is implemented using the tool “ee.Classifier.smileRandomForest()” and uses 6 input parameters (Table 3). The RF classifier was configured with 1500 decision trees, selected to ensure robust predictions and mitigate risks of overfitting. Key parameters included the number of variables per split, set to the default value (square root of total features), which ensures optimal feature selection at each tree node. The minimum leaf population was set to a default value of 1, allowing for precise distinctions between land cover classes. Additionally, the fraction of input samples bagged per tree was defined as 0.5, balancing model bias and variance. These parameters were established through preliminary testing, with 1500 trees demonstrating consistent stability and reliability across multiple classifications.

Regarding optimization methods, no advanced optimization techniques such as grid search or random search are employed in this study. The selected parameters are based on prior experience and common practices for remote sensing classification tasks. The parameters, particularly for the RF and SVM classifiers, are thus considered default or typical values for these models but can be adjusted if necessary to optimize performance further in future studies.

For both algorithms, to achieve higher classification accuracy, we also used the NDVI and NDBI spectral indices as input, similar to the study of Svoboda et al. (2022). These indices were used to train the models to better distinguish between vegetation and built-up areas.

To evaluate classification accuracy and determine a more reliable classifier, the accuracy assessment was performed separately for the 2017, 2019, and 2023 classification results and for the two classification algorithms used (SVM and RF). Training and testing data sets were established at an 80:20 ratio. The training data sets were used for model training, whereas the testing datasets were employed for validating the accuracy of the classifications. This split ensures that the classifiers are evaluated on unseen data, providing an unbiased estimate of their generalization performance. Cross-validation, in the form of this train/test split, is implemented for each year (2017, 2019, and 2023) to assess the classifiers’ ability to handle temporal variations. This method of splitting the data is widely used to ensure that the classifiers are not overfitting and to provide an objective measure of model performance. The random split, along with the confusion matrix generated for each year’s classification, allows for a clear evaluation of classifier accuracy and helps ensure that the results are reproducible across different datasets and time periods. The classifications’ accuracy was evaluated and expressed in terms of the error matrix, along with metrics such as overall accuracy, user’s accuracy, producer’s accuracy, and the Kappa coefficient. These metrics provide a comprehensive assessment of the classifier’s performance, ensuring that the model is both reliable and effective in distinguishing between different land cover classes.

## Results

### Downscaled LST using the relationship between spectral indices and LST

The LST prediction model was established using ordinary least squares linear regression and three predictors—NDVI, NDWI, and NDBI. Scatter plots between the pairs of variables, Landsat-8 LST, and each predictor revealed a significant correlation between the spectral indices and Landsat-8 LST in the Prešov city: a negative correlation with NDVI and positive correlations with NDBI and NDWI.

**Fig. 5:**
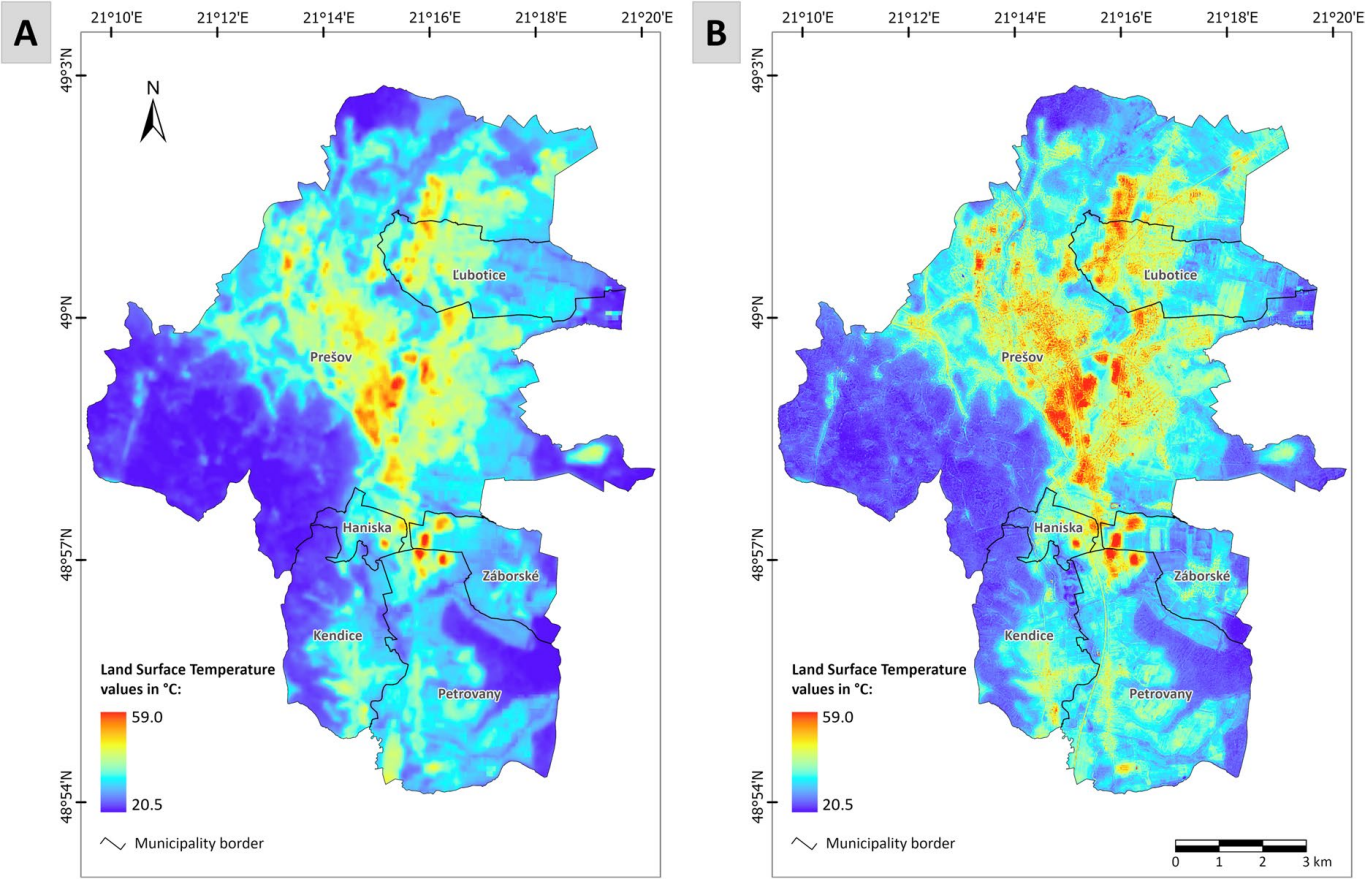
(**A**) Coarse land surface temperature (LST_c_) derived from Landsat-8 in spatial resolution of 30 m, (**B**) downscaled finer land surface temperature (LST_f_) in spatial resolution of 10 m, for the study area of the city of Prešov in 2023

The results show that there is a direct correlation between LST and NDBI (*r*_2017_ = 0.71, *r*_2019_ = 0.66, *r*_2023_ = 0.55) which indicates that LST increases with increasing proportion of built-up areas. This observation agrees with the multivariate analysis. An indirect relationship is observed and was found to be significant at *p* < 0.001 for all years analyzed between LST and NDVI (*r*_2017_ = − 0.73, *r*_2019_ = − 0.61, *r*_2023_ = − 0.85) and LST and NDWI (*r*_2017_ = − 0.72, *r*_2019_ = − 0.67, *r*_2023_ = − 0.60). Based on these results, it can be inferred that the correlation values suggest that NDBI, NDVI, and NDWI are good indicators for LST, which is aligned with the findings of Purio et al. (2022). Figure 5 shows a comparison of downscaling results for one of those years (2023)—LST_c_ in the original 30-m spatial resolution—before downscaling (Fig. 5A) and LST_f_ after downscaling in 10-m spatial resolution with clearly improved (sharpened) LST pattern (Fig. 5B). The multiple linear regression model incorporating NDVI, NDBI, and NDWI achieves a high coefficient of determination (*R*^2^ = 0.92, *p* < 0.001). Differences in the distribution of LST are closely associated with specific land cover (LC) categories. The LST patterns shown in Fig. 5 indicate the presence of the UHI phenomenon in the city. The lowest LST values in the study area are observed within a large forest park in the western part of Prešov, characterized by substantial vegetation cover and minimal human intervention. These vegetated areas provide natural cooling through shade and evapotranspiration, emphasizing their critical role in mitigating surface temperatures. Low-temperature values are also found in the southern part of the area with agricultural fields and along the Torysa River valley, where the combination of shrubland with trees and open areas of permanent grassland maintains relatively lower temperatures. In contrast, the highest values, sometimes reaching up to 50 °C are visible in the historical city core, areas of other urban fabrics on the outskirts of the city core and recently built road communications and industrial zones in the south of the city core. These areas are characterized by impermeable surfaces such as asphalt and concrete, which absorb and retain heat, playing a substantial role in the development of the UHI phenomenon. The replacement of vegetation and natural surfaces with such materials eliminates cooling processes and amplifies local warming, particularly in densely developed urban areas.

**Fig. 6:**
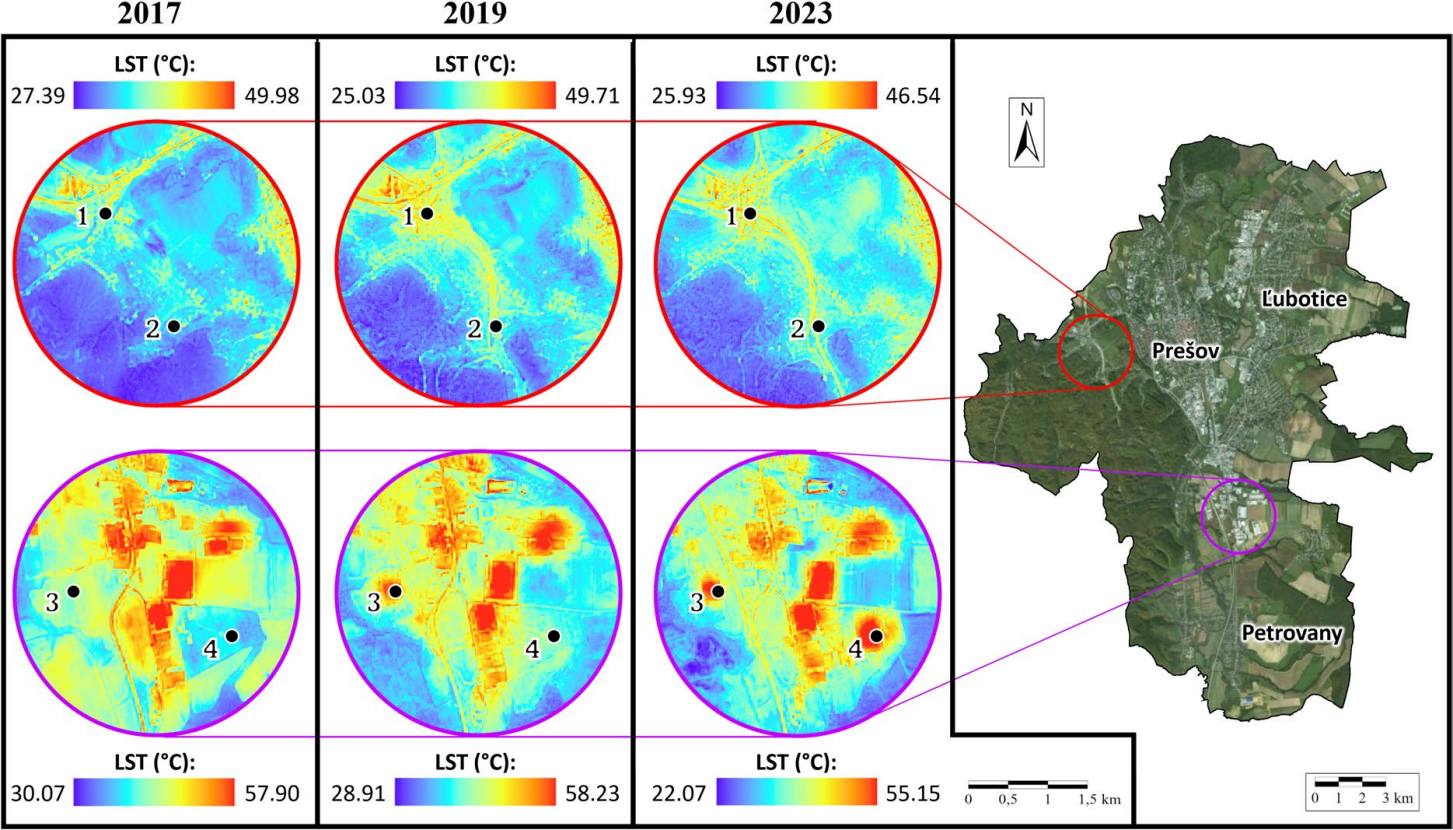
Sites 1–4 where significant changes in land surface temperature between the years 2017 and 2023 were detected. Source: land surface temperature maps: author’s processing, the background true color map (on the right) is © ESRI. “World Imagery.”

Differences in LST patterns between selected images in 2017, 2019, and 2023 were predominantly observed in suburban areas affected by the construction of a highway bypass and the development of new buildings in the industrial parks of Haniska and Petrovany. These selected sites were further analyzed using LST_f_. The location map is depicted in Fig. 6, with numbers 1–4 marking 4 sites where significant changes in LST were detected within the area of interest.

The corresponding changes in LSTs for these four sites are presented in Table 4. Based on the maps in Fig. 6 and the data presented in Table 4, we observe that in areas where LC has changed due to the expansion of built-up areas and roads, there is a corresponding increase in LST values. Localities 1 and 2 indicate sites located on newly constructed sections of the highway and expressway, where LST increased approximately 6–7 °C in 2023 (after construction) compared to 2017 (before construction). These increases are directly attributable to the materials used in road construction, such as asphalt, which has a low albedo and high heat retention capacity. The transformation of these areas from agricultural or natural land into impervious surfaces has altered the local microclimate by disrupting natural cooling processes, including evapotranspiration and vegetation-mediated cooling, thereby intensifying localized warming.

**Table 4:** Land surface temperature values for selected 4 sites in 2017, 2019, and 2023

Site	Change in LC	LST (°C)		
		2017	2019	2023
1	Road communication—expressway	36.7	40.3	42.5
2	Road communication—highway	35.3	38.8	42.9
3	Industrial zone—production building	38.1	47.7	48.1
4	Industrial zone—logistics center	35.0	36.9	51.9

An even greater difference in LSTs can be observed at sites 3 and 4, where new buildings have been constructed in the industrial areas of Haniska and Petrovany. At site 3, the LST value was 38 °C in 2017 (before the construction of the production building), and by 2023, this value had increased by approximately 10 to 48 °C (after the construction of the production building). Similarly, at site 4, the LST increased by approximately 17 °C after the construction of the logistics center, rising from 35 °C in 2017 to almost 52 °C in 2023. These changes underscore the thermal impact, where impervious surfaces such as concrete and metal roofs dominate the landscape.

The increase in LST values in these cases is primarily attributed to the alteration in LC and the nature of the newly developed surfaces, previously characterized by natural landscapes or agricultural use. These areas underwent significant transformation by 2023, with the introduction of impermeable artificial materials such as asphalt roads (sites 1–2) and buildings with metal roofs (sites 3–4). These changes highlight the profound implications of infrastructure growth and urbanization, as the replacement of natural vegetation with impervious surfaces not only absorbs and retains heat but also limits evaporative cooling, thereby intensifying the UHI effect in both urban and suburban areas (Zhang et al., 2017). This thermal imbalance directly affects the local microclimate, environment, and society, amplifying localized warming and its associated challenges.

Industrial zones (sites 3 and 4) exhibited an even more pronounced increase in LST, particularly at site 4, where the logistics center caused an increase of nearly 17 °C. Such a dramatic rise underscores the contribution of large impervious surfaces, such as concrete and metal roofs, to the UHI effect. These changes not only contribute to intensified local temperatures but may also have broader implications for air quality, biodiversity, and the thermal comfort of residents.

From an environmental perspective, the observed increase of LST emphasizes the need for a sensible urban planning that integrates sustainable measures. For instance, incorporating green infrastructure, such as tree planting, green roofs, and high-albedo materials, can help mitigate the UHI effect and promote a more resilient urban environment. Without such interventions, rapid urbanization may exacerbate energy demands for cooling and reduce the city’s adaptability to extreme climate events such as heatwaves.

### Image classification using machine learning algorithms—support vector machine (SVM) and random forest (RF)

**Fig. 7:**
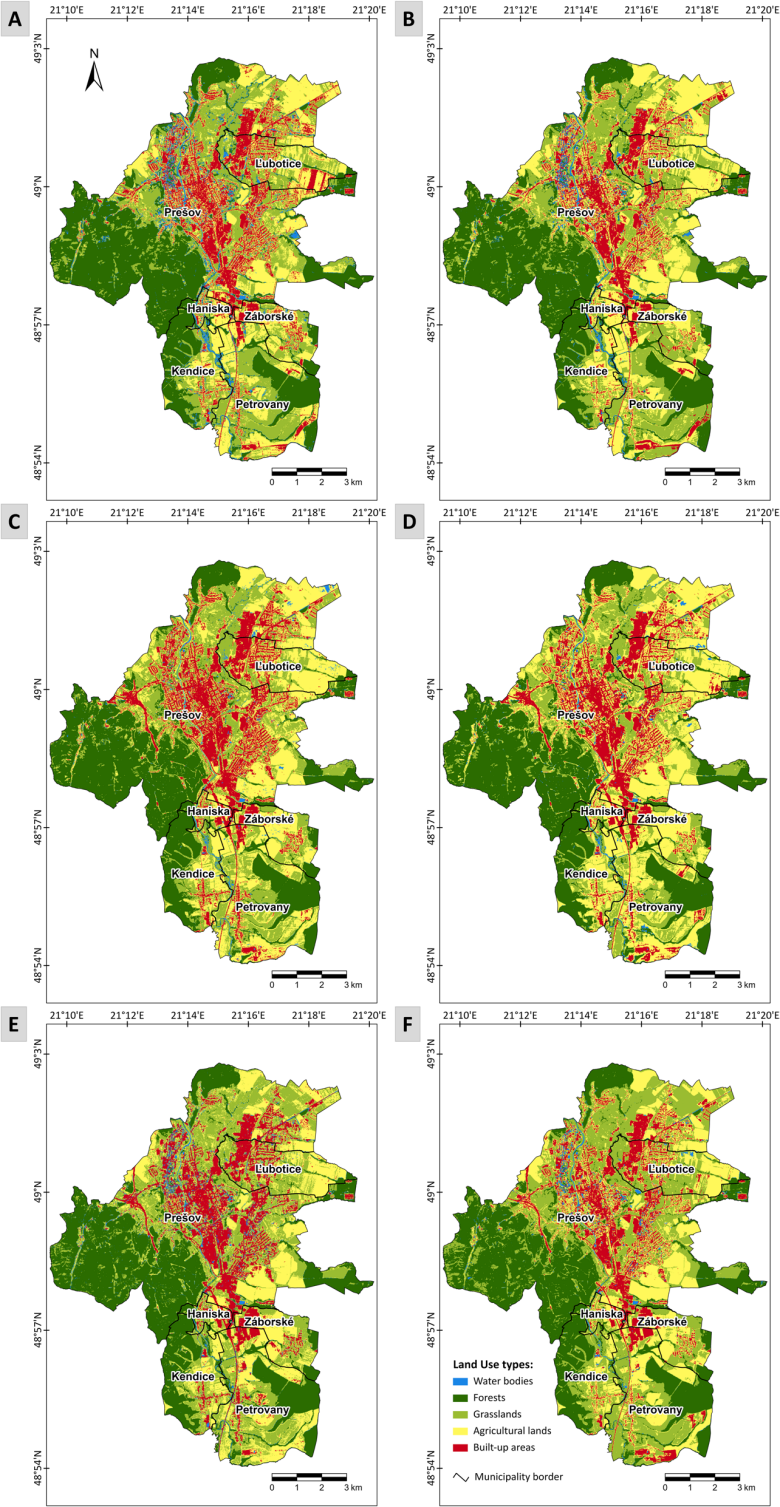
Supervised classification using support vector machine (SVM) and random forest (RF) classification algorithms: **A** SVM 2017, (**B**) RF 2017, (**C**) SVM 2019, (**D**) RF 2019, (**E**) SVM 2023, (**F**) RF 2023

The second part of our research was dedicated to the semi-automatic supervised classification of satellite imagery using two machine learning algorithms SVM and RF. Classification outputs using the SVM and RF algorithms are shown in Fig. 7. The classification performance of both algorithms was evaluated using overall accuracy, Kappa index, and both the producer’s and user’s accuracy for each land cover class. The accuracy assessments of both algorithms for the respective years 2017, 2019, and 2023 are displayed in Table 5.

**Table 5:** Accuracy assessment of image classifications using support vector machine and random forest algorithms for 2017, 2019, and 2023

	Support vector machine			Random forest		
	2017	2019	2023	2017	2019	2023
Overall accuracy	92.4%	92.2%	87.2%	93.2%	89.6%	91.5%
Kappa index	90.5%	90.2%	83.9%	91.5%	86.9%	89.2%
Producer’s accuracy						
Water bodies	96.0%	95.5%	81.8%	96.0%	95.2%	85.0%
Forests	96.3%	92.0%	100.0%	96.3%	88.5%	96.3%
Grasslands	100.0%	89.3%	84.6%	100.0%	92.3%	87.1%
Agricultural lands	76.9%	86.4%	73.9%	80.0%	81.8%	100.0%
Built-up areas	95.0%	100.0%	95.2%	95.2%	90.0%	91.3%
Users’ accuracy						
Water bodies	96.0%	91.3%	94.7%	96.0%	87.0%	89.5%
Forests	100.0%	100.0%	96.2%	100.0%	100.0%	100.0%
Grasslands	87.0%	96.2%	75.9%	87.0%	92.3%	93.1%
Agricultural lands	95.2%	86.4%	85.0%	95.2%	81.8%	80.0%
Built-up areas	82.6%	85.7%	87.0%	87.0%	85.7%	91.3%

Based on the visual assessment in Fig. 7, semiautomatic classification using SVM (Fig. 7A, C, E) and RF (Fig. B, D, F) machine learning algorithms demonstrated considerable success, although some instances of pixel misclassification were observed (e.g., built-up areas classified as water bodies, or agricultural land as built-up areas)

According to Table 5, the overall accuracy of the six classifications evaluated using the SVM and RF algorithms ranged from 87.2 to 93.2%. Satellite imagery from 2017 was classified with an accuracy of 92.4% by the SVM model and 93.2% accuracy by the RF model. In 2019, the SVM model had a reliability of 92.2%, slightly less than 3% more than the RF model. By 2023, the SVM model’s success rate had dropped to 87.2%, while the RF model’s success rate was approximately 91.5%. In terms of the Kappa index, SVM showed a decline over time, from 90.5% in 2017 to 83.9% in 2023. Conversely, RF demonstrated more consistent performance, with the Kappa index remaining stable between 91.5 and 89.2%. These results highlight the superior and more stable performance of the RF model across all years compared to SVM.

Looking at the producer’s accuracy, the models performed similarly for certain land cover classes. For water bodies, both models showed high producer’s accuracies, while forests were classified consistently well, particularly by RF, which maintained accuracy above 88.5% across all years. For grasslands, SVM performed well in 2017 (100%), but RF demonstrated more stable performance throughout the study period, with accuracy ranging from 87.1 to 92.3%. Agricultural land accuracy was higher with RF, ranging from 80 to 100%, while SVM showed some fluctuation, ranging from 73.9 to 86.4%. Built-up areas were classified well by both models, with RF achieving better accuracy in 2023 (91.3%).

In terms of user’s accuracy, both models performed well for water bodies, forests, and built-up areas, with RF performing slightly better for built-up areas in 2023 (91.3%). However, SVM saw a sharp drop in accuracy for grasslands in 2023 (75.9%), while RF remained stable throughout the years (92.3–93.1%). For agricultural land, both models showed similar accuracy, but RF’s accuracy dropped to 80% in 2023.

Several factors impacted the accuracy of image classification. Primarily, the spatial resolution of Sentinel-2 images played a significant role. The class of water bodies was frequently misclassified due to the difficulty in identifying the Torysa River and its tributaries in parts of the images. This challenge can be related to their width which is less than 10 m, the native resolution of the Sentinel-2 bands. The second factor concerns the radiometric characteristics of satellite scenes, particularly when they are median composites generated from satellite images over several months. This can result in minor inaccuracies in classification, such as with agricultural soils, because the varying stages of vegetation of individual crops can make them harder to distinguish from grasslands.

Based on the results achieved, we evaluated that the RF algorithm appeared to be more reliable, and therefore, in the further analysis, we exclusively used the classification results of this particular more reliable classifier (RF). Higher reliability of RF classification was also confirmed, for example, by studies by Chowdhury (2024) and Zafar et al. (2024).

### Spatiotemporal evaluation of changes in land cover and land surface temperature

**Fig. 8:**
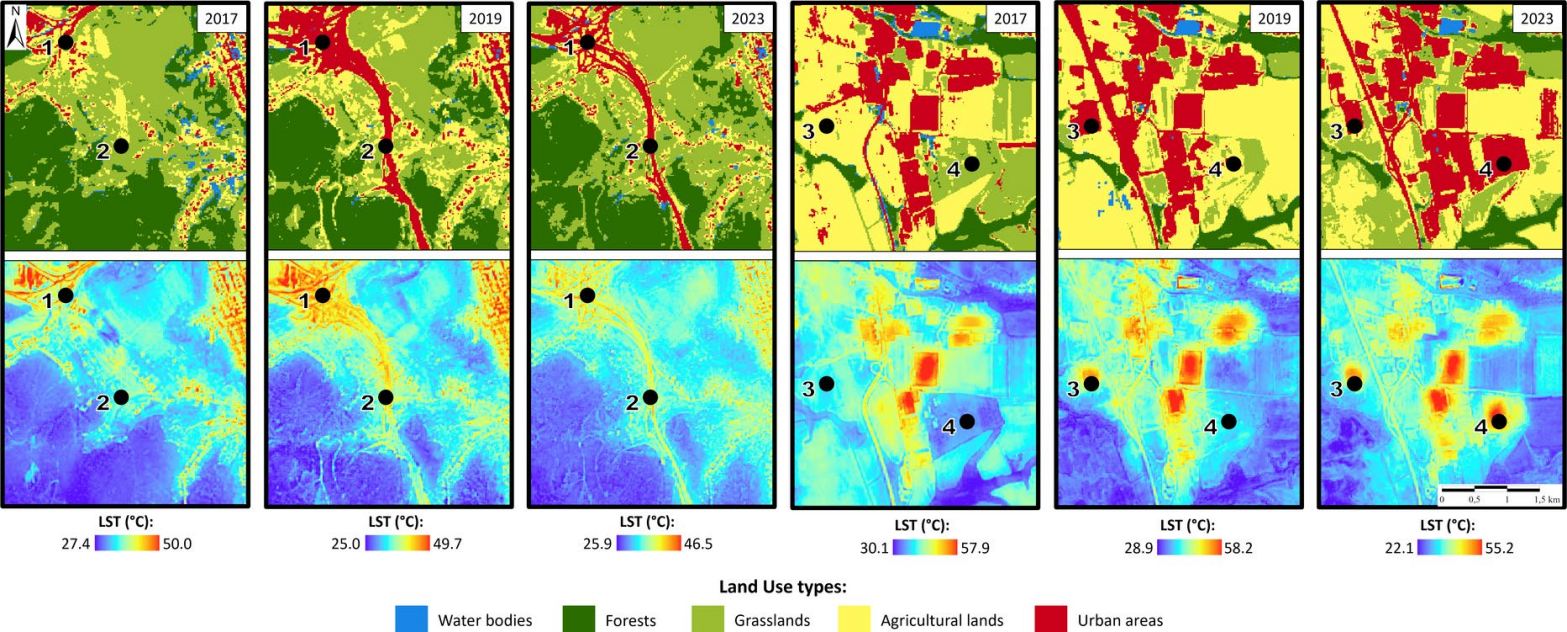
Land cover changes evaluated using supervised classification employing the random forest algorithm, along with the respective downscaled land surface temperature for the years 2017, 2019, and 2023 at sites 1–4

Spatiotemporal analysis was conducted using derived downscaled LST outputs in 10-m spatial resolution and the results of supervised classification by the RF classifier for the years 2017, 2019, and 2023. Figure 8 shows four localities within the area of interest, represented by image classification by the RF algorithm and the corresponding LST, highlighting the most significant changes in LC. The LST values for these sites are presented in Table 4.

Sites 1 and 2 depict areas where the highway bypass and tunnel were constructed between 2017 and 2019, leading to an increase in LST. In 2017, site 1 consisted of grassed areas and part of a garden area, with an LST value of approximately 37 °C. By 2019, during road construction, the LST increased to 40 °C, and by 2023, it had risen to nearly 43 °C. A similar trend is observed at site 2, where the LST value increased from 35 °C in 2017 to nearly 43 °C in 2023. Sites 3 and 4, which were previously agricultural land, have been transformed into built-up areas. At site 3, LSTs were approximately 38 °C in 2017. By 2019, the SPINEA, s.r.o. production building was constructed on this site, featuring a metal roof, metal facade, and concrete parking lot, resulting in an LST exceeding 48 °C by 2023. A similar transformation occurred at site 4, where agricultural land was replaced by an industrial zone—CTPark Prešov South, housing multiple companies. The entire area is paved with concrete surfaces and the buildings feature metal roofs and facades. This has led to an increase in LST values from 35 °C in 2017 to nearly 52 °C by 2023.

The areas surrounding both sites are exclusively agricultural lands without trees. Vegetation consists mainly of shrubs near local streams, although these were not clearly detected by our image classifications due to the spatial resolution. However, pixels depicting water bodies in the actual landscape exhibit noticeably lower LSTs compared to the adjacent banks, which are lined with stones.

### Data validation

**Fig. 9:**
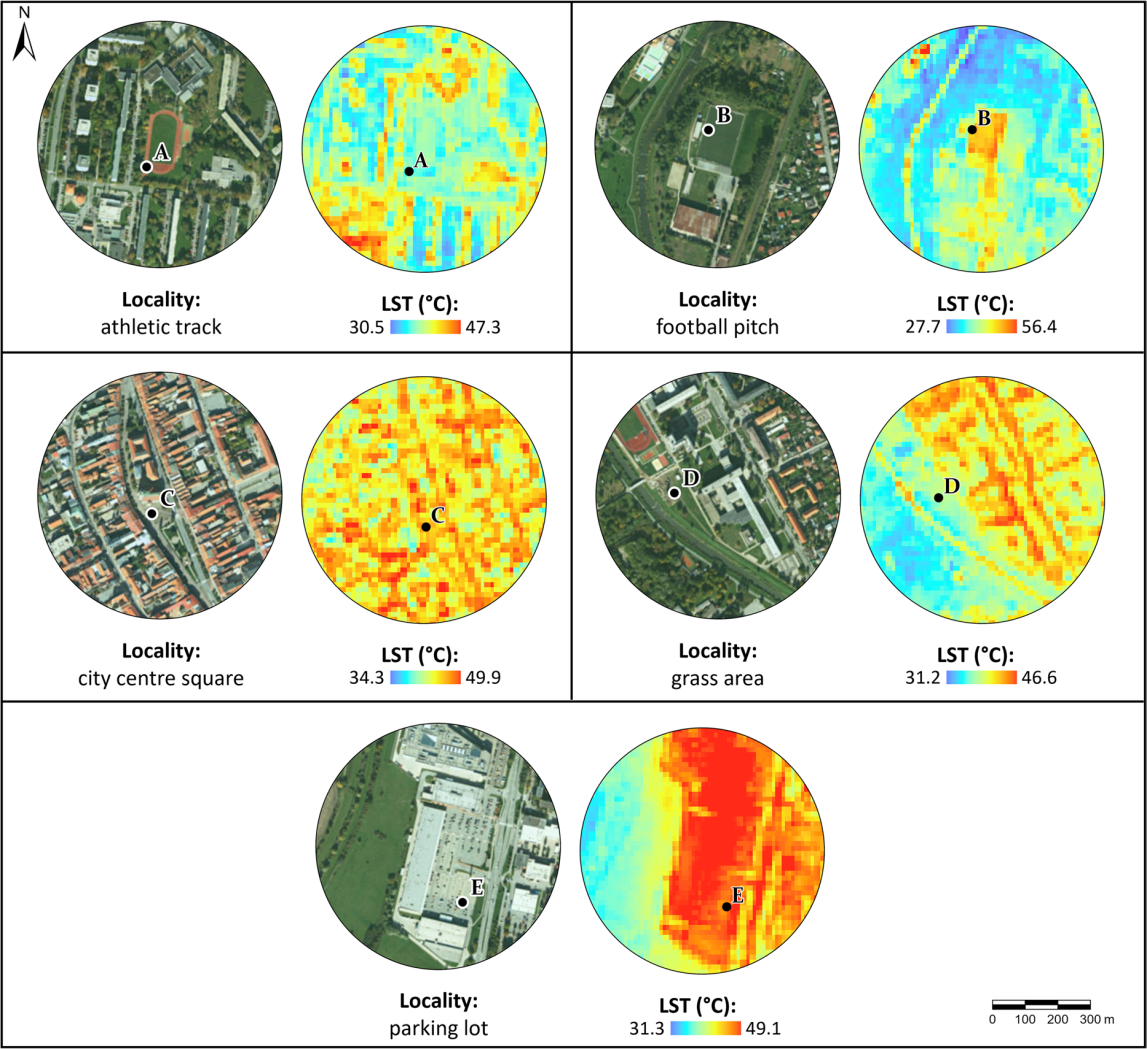
Localities **A**–**E** where the validation data were collected, the background images—true color and their corresponding land surface temperatures. The background true color map (on the left) is © ESRI. “World Imagery.”

To assess LSTs in the urban environment, validation data was collected using a data logger at five selected sites (A–E) within the urban area of Prešov. The sites and corresponding LST data are recorded in Fig. 9 and Table 6.

**Table 6:** Selected localities in Prešov, listing their kinetic surface temperatures measured by the Comet data logger (LST_dl_) and comparing them to the corresponding land surface temperature pixel value—30-m pixel from coarse Landsat-8/Landsat-9-derived land surface temperature (LST_c_) and downscaled—finer 10-m pixel of land surface temperature (LST_f_)

Locality	Characteristic	LST _dl_ (°C)			LST_c_ (°C) 09:26:16	Resid. LSTc-LST_dl 10:00_	LST_f_ (°C) 09:26:16	Resid. LST_f_ -LST_dl 10:00_
		07:00	10:00	13:00				
A	Tartan	27.8	32.3	-	35.9	3.6	34.1	1.8
B	Artificial grass	29.0	35.8	30.1	37.3	1.5	38.4	2.6
C	Stone paving	30.9	40.1	-	40.3	0.2	42.7	2.6
D	Natural grass	29.6	35.6	-	43.0	7.4	36.9	1.3
E	Concrete	31.3	39.5	35.4	37.1	− 2.4	45.0	5.5

Figure 9 shows that areas with higher LST values correspond to specific forms of urban fabric (continuous urban fabric in the city center, discontinuous urban fabric such as blocks of flats, commercial buildings, roads, parking lots, etc.). Conversely, pixels displaying lower LST values correspond to areas with vegetation, which likely contributes to cooling the surrounding environment.

Locality A is a tartan-covered athletic track located on the grounds of the elementary school grounds. Minimum LST values here hover around 30 °C, occurring in areas with dense tree vegetation. The maximum LST, reaching approximately 47 °C, is observed in the southwest part of the area, attributed to the light sheet metal roof of a department store. Locality B was located near a football field with artificial grass, adjacent to the Torysa River. Typically, water surfaces exhibit lower LST values, but here the river’s pixels show higher values likely due to lower spatial resolution. Along the nearby banks of the Torysa River, minimum LST values of around 27 °C were identified. This cooling effect is attributed to the widespread vegetation cover that includes grassy areas, meadows, and dense tree stands. In contrast, the maximum value of LST in this area was recorded near the swimming pool, where the surfaces are made of brick and stone pavements, reaching over 56 °C.

Locality C, in the city center, shows notably high LST values close to 50 °C. The minimum LST here is 34 °C, the highest among the studied localities, which we relate to dense urban fabric with red tin roofs and impermeable surfaces like stone streets and squares. Vegetation, such as trees in parks and along the main square, is less distinguishable in LST pixel values due to the low resolution of the satellite sensor, which primarily captures the thermal characteristics of built-up areas. Locality D is located within the southern park area near the University of Prešov, not far from the Torysa River. In particular, the central and eastern parts are densely built areas, with maximum LST values exceeding 46 °C. On the contrary, the western part of this section comprises forested areas where the minimum LST values hover around 31 °C.

Locality E encompasses the largest housing estate in the city, Sekčov, featuring shopping centers with metal roofs and extensive concrete parking lots. These surface characteristics are associated with maximum LST values approaching 50 °C. In contrast, the western area adjacent to the built-up zone shows a minimum LST of approximately 31 °C, characterized by long-term grassy areas interspersed with bushes.

Table 6 shows that the LST_dl_ values recorded by the handheld data logger (at 10:00 UTC) are consistently lower than the corresponding pixel values derived from satellite images LST_c_ (acquisition time, 09:26:16 UTC). This is primarily due to the higher accuracy and pointwise measurement capability of the data logger. The mean error (ME) values of 2.06 for LST_c_ and 2.76 for LST_f_ indicate that both models tend to overestimate the actual values. RMSE for LST_c_ (before downscaling) is 3.89, while for LST_f_ (after downscaling) it is 3.12. Here, LST_f_ performs better with lower RMSE, indicating smaller overall deviations from the ground-observed data (LST_dl_). The *R*^2^ values revealed a substantial difference in the correlation of the two datasets—LST_c_ and LST_f_ with ground-observed data. For LST_c_, the *R*^2^ value was 0.07, indicating a weak correlation between the predicted LST and the observed ground measurements, suggesting a poor predictive performance. In contrast, LST_f_ exhibits a significantly higher *R*^2^ value of 0.92 (*p* < 0.0001), indicating a strong and reliable correlation with ground-observed data. This demonstrates its strong capability in reconstructing the original LST observed by Landsat-8, as evidenced by a high Pearson correlation coefficient of 0.95.

In the morning at 7:00 a.m. under clear weather conditions, tartan recorded the lowest temperature at 27.8 °C, followed by artificial grass at 29.6 °C, and natural grass at 29.0 °C. The stone paving reached 30.9 °C, while the concrete had an LST of 31.3 °C. At 10:00 a.m., with no change in cloud cover, these temperatures rose as follows: tartan increased by less than 5 °C to 32.3 °C, both natural and artificial grasses increased by 6.8 °C to approximately 35.6 °C and 35.8 °C respectively. Stone paving and concrete showed the most significant increases in surface temperature, rising by almost 10 °C to around 40 °C.

The LST_dl_ values obtained from the data logger were compared with those derived from satellites at 30-m spatial resolution (before downscaling) and 10-m resolution (after downscaling) from Landsat-8 data acquired at 9:26:16 UTC. Even in the satellite-derived LST_f_ data, tartan showed the lowest value at 34.1 °C, followed by natural grass at approximately 36.9 °C, artificial grass at 38.4 °C, stone paving at 42.7 °C, and concrete that recorded the highest temperature at 45.0 °C.

Despite differences between the LST values obtained from the data logger and satellite data, there are similarities in the overall LST patterns observed with both methods. Both data sets show a consistent trend from the coldest to the warmest surface types, although the order of the LST data for concrete and stone paving changed after downscaling.

Our findings indicate that man-made surfaces, such as concrete and stone pavements, experience rapid heating, up to 10 °C in 3 h under sunny conditions. These surfaces are prevalent in urban areas, contributing to elevated LSTs and thus heating their surroundings. On the contrary, natural surfaces, such as vegetation, exhibit lower LSTs, which help cool their environment. Some natural grassy areas occasionally show slightly higher LST values, likely due to exposed soils from surface damage or increased solar energy exposure in the early morning (see Figs. 3D, 9). For instance, research conducted by Park et al. (2021) demonstrates that shaded and sun-exposed grass have distinct impacts on LST; shaded grass exhibits a cooling effect, whereas grass and low vegetation exposed to direct solar radiation are less efficient in mitigating land surface temperature. Additionally, the study by Song and Park (2014) emphasized that LST variations for vegetated areas were significantly influenced by measurement sites. These differences arose from the heterogeneous distribution of vegetation and non-vegetation zones or from variations in solar radiation absorption, which depended on the orientation of the leaves. Considering the prevalence of impermeable surfaces like concrete, asphalt, and stone in urban areas, the potential for the UHI phenomenon in the city is evident.

## Discussion

This study provides valuable insights into the understanding of UHI effects in medium-sized cities, particularly in Prešov, where such effects have not been extensively studied before. By enhancing the spatial resolution of satellite-derived LST data from 30 to 10 m, the research offers a more detailed view of temperature variations within the urban environment. This high-resolution mapping is crucial for identifying localized heat hotspots, which is essential for urban planning and mitigation strategies in rapidly urbanizing cities like Prešov. This contribution addresses a gap in existing UHI research, as studies focusing on medium-sized cities remain less common compared to larger urban centers (e.g., Cardoso et al., 2017; Oke, 1982; Santamouris, 2014).

Furthermore, this research builds on previous studies that explored the relationship between land cover and LST, but it goes further by applying advanced machine learning algorithms—SVM and RF—for more accurate classification of satellite imagery. The study highlights the effectiveness of these algorithms for LST downscaling in medium-sized cities like Prešov, demonstrating that the RF algorithm outperforms SVM in terms of overall accuracy and Kappa index. These findings are consistent with other studies (e.g., Chowdhury, 2024; Zafar et al., 2024; Zhang et al., 2023), which support the utility of RF in handling complex urban data.

The research also underscores the critical role of land cover transformation—specifically the conversion of natural and agricultural land into impervious surfaces—in amplifying UHI effects. This aligns with broader research on urbanization’s impact on local climates (e.g., Wang et al., 2024; Ul Moazzam, 2022). By examining LST changes over several years (2017 to 2023), this study provides a deeper understanding of how infrastructure development contributes to the intensification of UHI effects in Prešov. Additionally, the potential influence of seasonal variations on UHI effects is acknowledged, an area that remains under-explored in medium-sized cities. Further research could investigate how seasonal dynamics interact with land cover changes to influence UHI intensities, as seen in studies like those by Yuan et al. (2023), who explored seasonal variations in UHI effects across different land cover types in urban areas. Similarly, studies by Luo et al. (2024) examined the seasonal variation of the urban heat island intensity (UHII) in Shenzhen, finding that vegetation coverage significantly reduced UHII, with a stronger cooling effect in summer. The cooling effect was mainly driven by evapotranspiration and leaf area index (LAI).

In Prešov, the increase in LST due to urbanization—particularly the transformation of natural and agricultural land into impervious surfaces like roads and buildings—has exacerbated the UHI effect. This disrupts natural cooling processes, such as evapotranspiration and vegetation cooling, leading to localized warming. Seasonal variations further influence this effect, with urban areas experiencing more intense warming during warmer months due to the heat retention properties of impervious surfaces. During cooler months, these areas may maintain higher temperatures compared to vegetated zones, prolonging the UHI effect (Sun et al., 2019). These findings highlight the need for urban planning strategies that consider both land cover and seasonal dynamics, promoting green space preservation to mitigate UHI effects and enhance environmental resilience.

The study also found that the RF algorithm outperformed SVM in terms of overall accuracy across the years analyzed. RF demonstrated higher reliability, with an accuracy of 93.2% for 2017 compared to the SVM’s 92.4%. By 2023, SVM accuracy dropped to 87.2%, while RF remained stable at 91.5%. This suggests that RF is better suited for classifying complex, high-dimensional remote sensing data. RF’s robustness to noise and overfitting, due to its use of multiple decision trees, leads to improved classification accuracy (Liu et al., 2022; Pelletier et al., 2016). In contrast, SVM can struggle with high-dimensional data, as its performance is sensitive to kernel selection and hyperparameter tuning (Mountrakis et al., 2011; Xie et al., 2021, 2024). Previous research conducted in the studies of, e.g., Chowdhury (2024) and Urso et al. (2023) has similarly found RF to be more effective for handling complex remote sensing data. These findings further reinforce the reliability of RF for urban climate studies and environmental monitoring.

### Implications for urban planning and methodology application

The findings of this study underscore the need for comprehensive urban planning strategies in medium-sized cities, such as Prešov, facing urbanization and its associated impacts. The significant increase in LST resulting from the conversion of natural landscapes and agricultural land into impervious surfaces has intensified the UHI effect. This urbanization disrupts natural cooling processes like evapotranspiration and reduces the cooling effects of vegetation, leading to localized warming. In response, urban planning efforts in such cities should prioritize the creation and preservation of green spaces, the integration of permeable surfaces, the implementation of cool roofs, and more sustainable building materials. These mitigation strategies are crucial for reducing the negative effects of the UHI, such as increased energy demand and public health risks, and are increasingly being adopted in cities worldwide as part of climate adaptation initiatives (Almashhour et al., 2024; O´Malley et al., 2015).

The semi-automatic classification methodology employed in this study offers significant potential for application to other environmental variables beyond LST, such as monitoring vegetation changes, land use shifts, and air quality. This approach can be adapted to different geographic locations, enabling it to assess the impacts of urbanization on local microclimates and provide valuable insights for urban planning. Moreover, this method is easily scalable, as it relies on satellite imagery and machine learning algorithms that can be applied in various regions, making it a versatile tool for assessing environmental dynamics globally. By utilizing this methodology in different settings, cities worldwide can better understand and mitigate the environmental consequences of urban growth, fostering more sustainable development practices.

### Limitations

This study aimed to enhance the spatial resolution of satellite-derived LST from 30 to 10 m. However, such downscaling inherently introduces uncertainties due to factors like atmospheric interference, sensor calibration errors, and the limitations of the satellite’s spatial and radiometric resolution. One approach to mitigate these uncertainties could involve incorporating in situ LST data from meteorological stations. However, integrating ground-based measurements is beyond the scope of this study and presents a potential area for future research to improve the accuracy of LST estimation.

Additionally, this study focused exclusively on LST, but a more comprehensive analysis could benefit from incorporating additional environmental variables, such as 2-m air temperature and humidity, which play a critical role in urban heat dynamics and public health impacts (Chen et al., 2023; Good et al., 2017). Since these variables cannot be directly derived from satellite imagery, their integration would require innovative data collection techniques, such as the use of ground-based lidar systems or dedicated temperature-monitoring campaigns, to provide a more holistic understanding of the urban microclimate.

Finally, it is important to note that satellite-based LST measurements are typically obtained under clear sky conditions, which may limit their representativeness in areas affected by persistent cloud cover or other atmospheric disturbances. To address this limitation, future studies could explore alternative downscaling methods that do not rely exclusively on satellite-derived LST, such as data fusion techniques, which could improve the robustness and applicability of LST measurements across varying atmospheric conditions.

### Future research

Future studies should expand upon the findings of this research by exploring the long-term effects of urbanization on the UHI phenomenon, considering seasonal variations. While this study focused on the impact of LC change on LST in Prešov, it would be valuable to further investigate how different urban forms, such as compact versus sprawling cities, influence UHI intensity. Future research could also examine the effects of various mitigation strategies, such as the incorporation of green roofs or urban parks, in reducing LST and enhancing environmental resilience.

Additionally, applying the semi-automatic classification methodology to assess other environmental variables, such as air quality or vegetation cover, could provide a more comprehensive understanding of how land cover changes influence urban microclimates and public health. Expanding this methodology to other medium-sized cities, both within Slovakia and globally, would further validate its broader applicability and generalizability.

Incorporating climate change scenarios into future studies is also crucial, as it would enable the evaluation of how future development patterns may exacerbate UHI effects and impact urban heat resilience. Long-term studies, including future climate predictions, could offer valuable insights for urban planners and policymakers aiming to develop sustainable, climate-resilient cities.

Future research will focus on thoroughly verifying the obtained results and applying the developed method to other medium-sized cities. Another key goal will be to enhance the classification technique for use outside the EU by incorporating global land cover databases, such as the Copernicus Global Land Cover Layers (Buchhorn et al., 2020) and ESA WorldCover (Zanaga et al., 2022). This expansion could potentially lead to even more accurate results, contributing to the broader application and reliability of the method in diverse geographic contexts.

## Conclusions

The effect of surface urban heat island (UHI) and the increase in land surface temperature (LST) concerns not only large cities but also smaller cities like Prešov in Slovakia with about 100,000 inhabitants. The main objective of this research was to analyze the spatial distribution and temporal changes of LSTs in the city of Prešov, addressing a gap in the existing literature on UHI effects in medium-sized cities. While this study focuses on Prešov, it contributes to the broader understanding of UHI effects by highlighting the role of land cover changes, the value of high-resolution LST geodata, and the potential of machine learning techniques. These findings provide useful insights into UHI and LST dynamics, offering implications for urban planning and mitigation strategies in cities of similar size.

This analysis used a downscaling technique based on a multiple linear regression model, which implemented satellite observations from the Landsat-8, Landsat-9, and Sentinel-2 missions. A significant correlation was observed between spectral indices NDBI, NDVI, NDWI, and LST that were leveraged to downscale the native Landsat thermal imagery of 30-m spatial resolution to a finer 10-m resolution, using a multiple linear regression model. This improved resolution allowed for a more precise identification of changes in LST during the years 2017, 2019, and 2023.

Evaluation of the impact of atmospheric conditions affecting LST downscaling results could serve as motivation for future research. However, capturing the complexities of these effects would require high-density in situ temperature measurements or airborne thermal spectrometry, which could enhance the accuracy and reliability of urban heat island models and contribute to more effective mitigation strategies.

Furthermore, two machine learning algorithms, SVM and RF, were employed to classify images and assess the influence of varying LC types and changes throughout the years 2017, 2019, and 2023 on the city’s LST patterns. Evaluation of LC changes using the RF classification algorithm revealed higher accuracy compared to SVM—RF outperformed SVM by 0.8% in 2017 and 4.3% in 2023. The results show that over recent years, a previously natural landscape has been extensively altered, primarily in suburban areas that cause the increase of LSTs, thus contributing to the emergence and intensification of the UHI phenomenon. These changes were mainly driven by the construction of a highway bypass and the expansion of industrial zones. It was observed that impervious man-made surfaces, such as concrete and stone pavements, experience rapid heating, up to 10 °C in 3 h, subsequently warming the surrounding areas.

Therefore, strategic urban planning and development are essential to promote sustainable expansion and protect urban greenery, ultimately improving the quality of life of city residents. These findings can inform urban planning and mitigation strategies in similar cities facing rapid urbanization, while also emphasizing the role of machine learning in enhancing environmental monitoring and urban climate assessments. This approach can be applied not only locally but also globally, contributing to more effective and data-driven urban climate management.

## Data Availability

No datasets were generated or analysed during the current study.
